# Subtype recognition and identification of a prognosis model characterized by antibody-dependent cell phagocytosis-related genes in breast cancer

**DOI:** 10.18632/aging.205575

**Published:** 2024-02-22

**Authors:** Li Wang, Menghan Li, Hongyu Yang, Fenghuan Dai, Ning Xie, Linhui Li, Meiying Zhu, Ran Ding

**Affiliations:** 1 Department of Breast Cancer, Tianjin Medical University Cancer Institute and Hospital, National Clinical Research Center for Cancer, Key Laboratory of Cancer Prevention and Therapy, Tianjin’s Clinical Research Center for Cancer, Key Laboratory of Breast Cancer Prevention and Therapy, Tianjin Medical University, Ministry of Education, Tianjin 300060, China; 2 Acupuncture-Moxibustion Clinical Department, First Teaching Hospital of Tianjin University of Traditional Chinese Medicine, National Clinical Research Center for Chinese Medicine Acupuncture and Moxibustion, Tianjin 300381, China; 3 Graduate School, Tianjin University of Traditional Chinese Medicine, Tianjin 300381, China; 4 Department of Oncology, First Teaching Hospital of Tianjin University of Traditional Chinese Medicine, Tianjin 300381, China; 5 School of Biomedical Sciences and Engineering, South China University of Technology, Guangzhou International Campus, Guangzhou 511442, China

**Keywords:** antibody-dependent cell phagocytosis, breast cancer, immune checkpoint inhibition, prognostic biomarker, tumor microenvironment

## Abstract

Background: Breast cancer (BC) is a heterogeneous tumor with a variety of etiology and clinical features. Antibody-dependent cell phagocytosis (ADCP) is the last step of immune checkpoint inhibition (ICI), and macrophages detect and recognize tumor cells, then destroy and engulf tumor cells. Despite the large number, negative regulators that inhibit phagocytic activity are still a key obstacle to the full efficacy of ICI.

Patients and methods: An ADCP-related risk score prognostic model for risk stratification as well as prognosis prediction was established in the Cancer Genome Atlas (TCGA) cohort. The predictive value of ADCP risk score in prognosis and immunotherapy was also further validated in the TCGA along with International Cancer Genome Consortium cohorts. To promote the clinical application of the risk score, a nomogram was established, with its effectiveness verified by different methods.

Results: In this study, the genes collected from previous studies were defined as ADCP-related genes. In BC patients, two ADCP-related subtypes were identified. The immune characteristics and prognostic stratification were significant different between them.

Conclusions: We identified two subtypes associated with ADCP gene expression in breast cancer. They have significant differences in immune cells, molecular functions, HLA family genes, immune scores, stromal scores, and inflammatory gene expression, which have important guiding significance for the selection of clinical treatment methods. At the same time, we constructed a risk model based on ADCP, and the risk score can be used as a good indicator of prognosis, providing potential therapeutic advantages for chemotherapy and immunotherapy, thus helping the clinical decision-making of BC patients.

## INTRODUCTION

The complete antibody of human immunoglobulin (IgG) consists of an antigen-binding fragment (Fab) and a crystal fragment (Fc) that binds to the Fcγ receptor. In the constant region of IgG1, IgG2, IgG3 and IgG4, IgG1 exhibits the highest FcγR binding affinity compared to IgG3, IgG2 and IgG4, particularly in the subclasses characterized by different hinge and CH2 domains [1]. The Fc region binds to FcγR on the surface of immune cells, and antibodies can cause some effector cells to produce strong cytotoxic effects [2]. Therefore, different subtypes possess distinct effector functions, such as ADCP.

Antibody-dependent cell phagocytosis (ADCP) of tumor is a kind of antibody that binds to target cells (such as tumor cells) and effector cells (such as macrophages) at the same time, resulting in the phagocytosis of target cells by effector cells [3]. After phagocytosis, target cells are digested and degraded in effector cells by acidification. ADCP is considered to be the main MOA for a variety of biological drugs such as anti-CD20, CD38, EGFR, HER-2 [4]. At the same time, macrophage-mediated tumor cell phagocytosis by using anti-CD47 antibodies to block the anti-phagocytic CD47-SIRPa interaction has shown promise in preclinical xenotransplantation of various human malignancies [5]. The satisfactory anti-cancer effect of ADCP depends on early antibody administration, and drug resistance develops with cancer progression [6].

BC is a heterogeneous tumor with a variety of etiology and clinical presentations. Studies have revealed the presence of various subtypes involved in the development and progression of BC [7]. Our research introduces the research status of breast cancer subtypes in detail and identifies new subtypes by means of machine learning [8], which opens up new fields for breast cancer research.

A large number of studies have proved the importance of ADCP in tumor treatment. In this study, we determined the ADCP-related subtypes of breast cancer, and comprehensively discussed the significance of ADCP in multi-omics. It provides an important basis for clinical precision treatment.

In this study, genes defined as ADCP-related genes were collected from previous studies. In BC patients, two ADCP-related subtypes were identified. The immune characteristics as well as prognostic stratification were significant different between the two ADCP-related subtypes. Based on these findings, it can be inferred that there may be a significant relationship between ADCP in BC and TME (tumor microenvironment). Risk score could be used as a good indicator for prognosis, potential in offering therapeutic advantages for chemotherapy as well as immunotherapy, thereby aiding in the clinical decision-making process for BC patients.

## MATERIALS AND METHODS

### Data download and processing

Study cohorts A and B were based on data obtained from two publicly available datasets that derived from The Cancer Genome Atlas (TCGA, https://portal.gdc.cancer.gov/) and Molecular Taxonomy of Breast Cancer International Consortium (METABRIC, https://www.cbioportal.org/study/summary?id=brca_metabric) databases, respectively [9, 10]. Cohort A consisted of 103 paracancerous samples and 903 breast cancer (BC) samples, which were used for gene expression profiling. On the other hand, cohort B included 154 paracancerous samples and 1826 BC samples for gene expression profiling. In order to evaluate the reliability of the prognostic model constructed in this study, cohort A was designated as the training set, while cohort B served as the validation set. This division allowed us to train the model on one dataset and validate its performance on an independent dataset.

### Identification of the survival-related ADCP genes

To evaluate the prognostic significance of ADCP genes, Cox regression analysis was further adopted to evaluate the relationship of each gene with survival status in the TCGA cohort. To mitigate the risk of overlooking potentially important genes, a truncated p-value of 0.05 was set as a threshold, and a total of 130 survival-related genes were identified for further analysis.

### Consensus clustering analysis of ADCP genes

The unsupervised clustering ‘Pam’ method was applied to the identification of different molecular subtypes based on survival-related ADCP gene expression [11]. The R package Consensus Cluster Plus performs this process and determines the number of clusters in Queue A, with 1000 times repeated to make sure classification stability [12]. Principal component analysis (PCA) was employed to illustrate the distribution differences. To evaluate the clinical significance of ADCP subtypes, their relationship with prognosis and other clinicopathological features (including age, stage and cancer subtypes) were examined. In different cohorts, the overall survival (OS) of different clusters was compared using the Kaplan-Meier (K-M) survival chart, with P ≤ 0.05 indicating statistical significance. Besides, the relationship between ADCP subtypes and other clinical variables was visualized using the Sankey plot and drawn using the ggalluvial R package [13]. The heat maps of different ADCP gene expression patterns were plotted by the pheatmap R package [14].

### Identification and verification of the key ADCP genes

Based on the TCGA, the ‘limma’ R package was adopted to identify the differentially expressed genes (DEGs) between group1 and group2 [15]. The fold change cut off was 1, and the adjusted p value was less than 0.05. We used the glmnet R package in the TCGA cohort to use the LASSO Cox regression model to screen out the best prognostic biomarkers in 130 ADCP genes [16]. A total of 39 ADCP genes with non-zero coefficients were screened by 10-fold cross-validation. We overlapped the ADCP genes screened by DEGs and LASSO regression to screen out the key ADCP genes. Risk scoring formula:

$$\text{Risk}\;\text{score}=\sum_{\text{i}=1}^{\text{n}}\text{coefi}*\text{id}$$ (1)

Where Coefi represents the coefficient and id represents the normalized count for each gene.

After constructing the risk score according to the LASSO regression model, the risk grouping and group were visualized through the Sankey diagram using the ggalluvial R package according to the survival situation.

Univariate Cox regression together with K-M plot were used to analyze the prognostic significance of key ADCP genes. P-values less than 0.05 indicated statistical significance. Subsequently, multivariate Cox regression model was utilized to explore whether ADCP genes could be combined with other clinicopathological features as independent prognostic factors.

### Correlation of the ADCP genes with copy-number alterations and immune traits

Gene Set Cancer Analysis (GSCA) is a multi-omics online analysis tool based on TCGA data. It was used to analyze the expression and prognosis of pan-oncogenes in key ADCP genes (http://bioinfo.life.hust.edu.cn/web/GSCALite/) [17]. Results are displayed in the bubble plot by the ‘ggplot2’ package [18]. To analyze the expression characteristics of key ADCP genes in tumor and ADCP, t-test algorithm was used to compare their expression levels in different risk scores, groups and tumor groups in cohort A, respectively. The CAN (copy-number alterations) ratio as well as methylation level (β value) of key ADCP genes among TCGA-BC samples were verified by GSCA online tool. As a convenient online tool, TISIDB provides convenient access to correlation between genes and immune traits, such as lymphocytes, immunomodulators, and chemokines [19]. The scatter plots of ADCP genes with several tumor-infiltrating lymphocytes (TIL), immunosuppressants and immunostimulants were downloaded from TISIDB.

### Construction and verification of risk model

For each patient, the risk score was predicted by the ‘predict’ function in the survival R package [20]. Patients were then divided into high- and low-risk groups according to the median value. To test the accuracy of the Cox regression model, C-indexes as well as receiver operating characteristic curves (ROC) were adopted. Prognostic factors, including C-indexes representing risk score, were visualized using histogram. ROC analysis was performed by the ‘pROC’ package [21], with the area under the curve (AUC) values calculated and compared to evaluate the performance of the model. In addition, the K-M survival analyses were conducted for risk scores. Based on training and test datasets, 1-, 3- and 5-year ROC curve were plotted using the R package timeROC [22].

### Nomogram construction

To further investigate the prognostic significance of ADCP genes, the TCGA datasets were utilized to generate risk plots between high- and low-risk groups. To improve the predictive accuracy, a nomogram incorporating TNM stage, Cancer type and group was developed using survival and rms package [23]. To demonstrate the consistency between the predicted 1-, 3- and 5-year endpoint events and the corresponding actual outcomes, calibration plots were generated using rms package.

### Evaluation of drug sensitivity

To investigate the molecular characteristics associated with drug sensitivity/resistance, the Genomics of Drug Sensitivity in Cancer (GSDC) database (https://www.cancerrxgene.org/) [24], a public resource for identifying cancer cell biomarkers, was utilized. The pRRophetic package was adopted to determine the sensitivity data of two BC groups towards various drugs [25]. Furthermore, the drug sensitivity of BC patients with different phenotypes was predicted from gene expression data.

### Quantitative reverse transcriptase PCR (qRT-PCR)

The MCF-10A (human breast cancer paracancerous) and MDA-MB-453 (human breast cancer) cell lines were obtained (Shangcheng North Na Chuanglian Biotechnology Co., Ltd.). Control groups were created by combining MCF-10A with MDA-MB-453 cells to compare the expression of DEFB1, SIAH2, and SYT1 between normal and breast cancer cell lines. Total RNA was extracted using the Redzol kit (Beijing SBS Gene Technology Co., Ltd.), and qRT-PCR was performed using the SYBR® Premix Ex Taq™ II Kit. The relative mRNA expression levels were calculated using the 2^−ΔΔCt^ method, with β-actin as the internal reference gene. The forward primer sequences were as follows: (1) DEFB1: F-5′-CTCAGGTGGTAACTTTCTCA-3′; (2) SIAH2: F-5′-CACTTGACAGGCTGTTGCAC-3′; (3) SYT1: F-5′-CGCTTCGGCAGCACATATACTAAAATTGGAAC-3′. (4) GAPDH: F-5′-GAGTCAACGGATTTGGTCGT-3′. The reverse primer sequences were as follows: (1) DEFB1: R-5′-AAGCACTCCGGGTGATTCAG-3′; (2) SIAH2: R-5′-AAGCACTCCGGGTGATTCAG-3′; (3) SYT1: R-5′-TTGGTCAGCACAGATCATCG-3′. (4) GAPDH: R-5′-GATCTCGCTCCTGGAAGATG-3′.

### Western blot (WB)

The WB analysis was performed following previously established protocols and the manufacturer’s recommendations. The concentrations of extracted proteins were determined using the Bradford method. Proteins were separated on a 10% SDS-polyacrylamide gel, transferred to PVDF membranes, and blocked with 5% skimmed milk. Primary antibodies against DEFB1 (1:500; catalog number ab115813; Abcam), SIAH2 (1:500; catalog number ab31234; Abcam), SYT1 (1:500; catalog number ab302627; Abcam), and GAPDH (1:500; catalog number ab181602; Abcam) were incubated with the membranes overnight at 4° C. Afterward, HRP-labeled secondary antibody was added and the membranes were washed with TBST. WB analysis was conducted using chemiluminescence, and the protein bands were visualized using a film.

### Statistical analysis

Statistical analyses were conducted using R software (version 4.2.1) [26]. A two-sided P-value less than 0.05 was considered statistically significant. The K-M survival curve was constructed using the survival curve function ‘ggsurvplot’ in the R package survminer (0.4.2 version) [27], and the bilateral log-rank test was adopted to estimate the difference in OS between different groups. The cut-off value of ADCP genes, differentiating the high-risk group from the low-risk group, was determined as the median or calculated using the surv cutpoint function implemented in the R package survminer (0.4.2 version) [28]. The LASSO Cox regression model was used to identify the ADCP genes that exhibited a significant association with OS, and multivariate Cox regression analysis was performed using the identified ADCP genes to calculate hazard ratios (HR), 95 % confidence intervals (CI), and corresponding p-values to construct ADCP genes. Additionally, multivariate Cox regression analysis was used to evaluate the independent prognostic value of clinical indicators, including the ADCP genes, and a nomogram visual risk prediction map was formed after scoring each factor using the rms package of R software. To evaluate the consistency of nomogram, the calibration plot was generated using the rms package of R software [29].

### Availability of data and material

Publicly available datasets were analyzed in this study. These data can be found here: https://portal.gdc.cancer.gov/, https://www.cbioportal.org/study/summary?id=brca_metabric. The names of the repository/repositories and accession number(s) can be found in the article/Supplementary Material.

### Consent for publication

All authors approved the publication of the article.

## RESULTS

### Survival-related ADCP gene

To investigate the prognostic significance of ADCP genes, a gene signature was established using data from cohort A. The workflow for the construction of gene signature is depicted in Supplementary Table 1. Based on univariate Cox regression, 130 prognostic genes were screened out (p-value < 0.01, Supplementary Table 1).

### Consensus clustering analysis of ADCP genes

**Figure 1:**
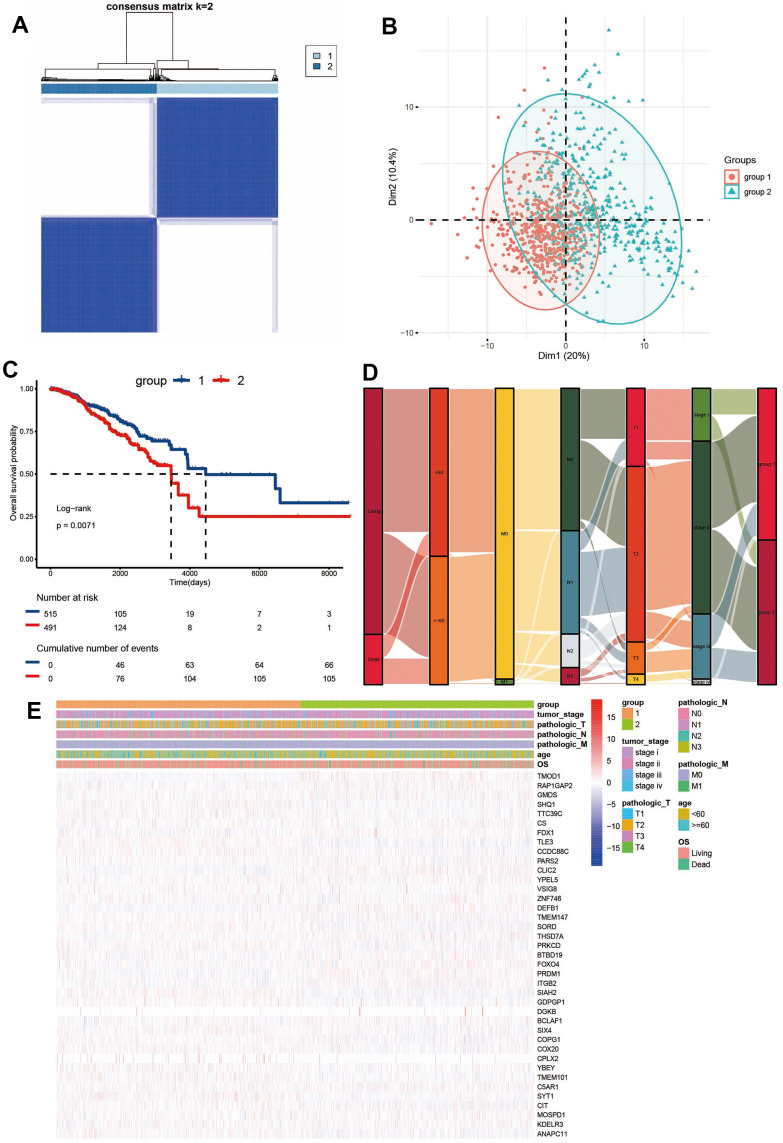
**Consensus clustering of ADCP-related genes in BC.** (**A**) The consensus matrices and (**B**) PCA analysis were performed to assess the stability of clustering and explore coagulation subtypes. (**C**) Kaplan-Meier curves and (**D**) an alluvial diagram demonstrated the association between ADCP groups, tumor stage, age, grade, and survival state. (**E**) Unsupervised clustering of all coagulation-related genes in TCGA cohorts.

Based on the expression levels of survival-related ADCP genes in the TCGA database, two distinct regulatory patterns were identified using an unsupervised clustering method. A total of 515 cases were classified into ADCP-related cluster 1 and 491 cases were classified into ADCP-related cluster 2 (Figure 1A). PCA analysis demonstrated that the patients can be classified into two distinct parts, providing further evidence of the existence of two significantly different subtypes (Figure 1B). To evaluate the survival difference between the two clusters, an analysis was conducted on a diverse range of patients from a variety of cohort A (TCGA) with complete survival information were analyzed. Subsequently, the relationship of these two clusters with various clinical features were analyzed (Figure 1D). The survival advantage of cluster 1 was higher compared to that of cluster 2. In cluster 1, the proportion of age less than 60, M0, N0-1, T1-2, Stagei-ii was higher. These suggest that ADCP genes may affect tumor development through some potential mechanisms. The heat map depicts the transcriptomic characteristics of different expressed ADCP genes in the two ADCP subtypes (Figure 1E).

### The immune landscape of ADCP subtypes

**Figure 2:**
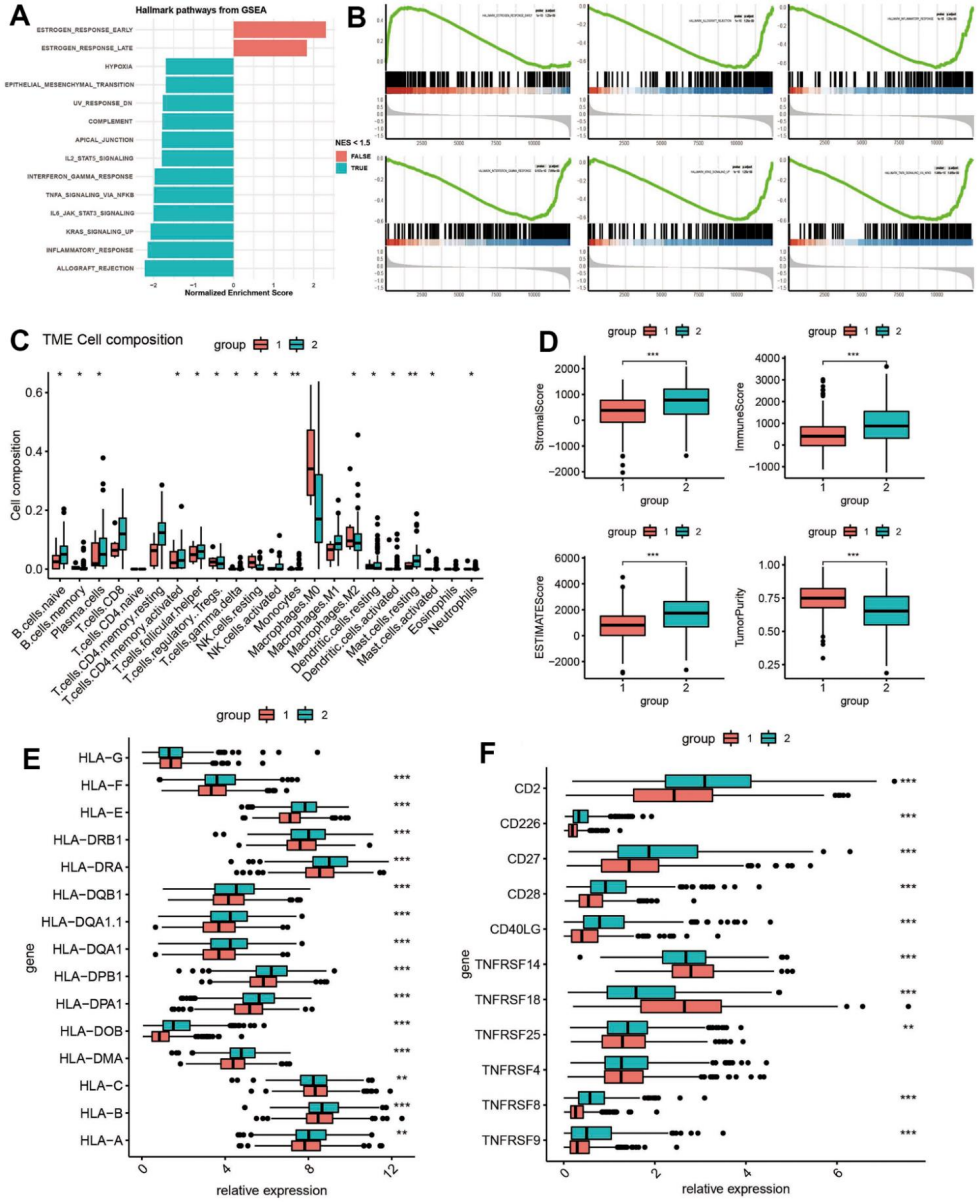
**Clinical significance and immune landscape of ADCP groups in the TCGA cohort.** (**A**) GSVA and Gene Set Enrichment Analysis (GSEA) revealed the activation or inhibition of biological pathways and (**B**) significant enrichment in immune-associated processes. (**C**) Immune cell infiltration, (**D**) stromal and immune scores, and gene expression of HLA and MHC gene sets were analyzed between ADCP groups. (**E**) The expression box plot of HLA family genes between the two subtypes. (**F**) The expression box plot of inflammatory genes between the two subtypes. Statistical significance at the level of * <0.05, ** <0.01, and *** <0.001.

**Figure 3:**
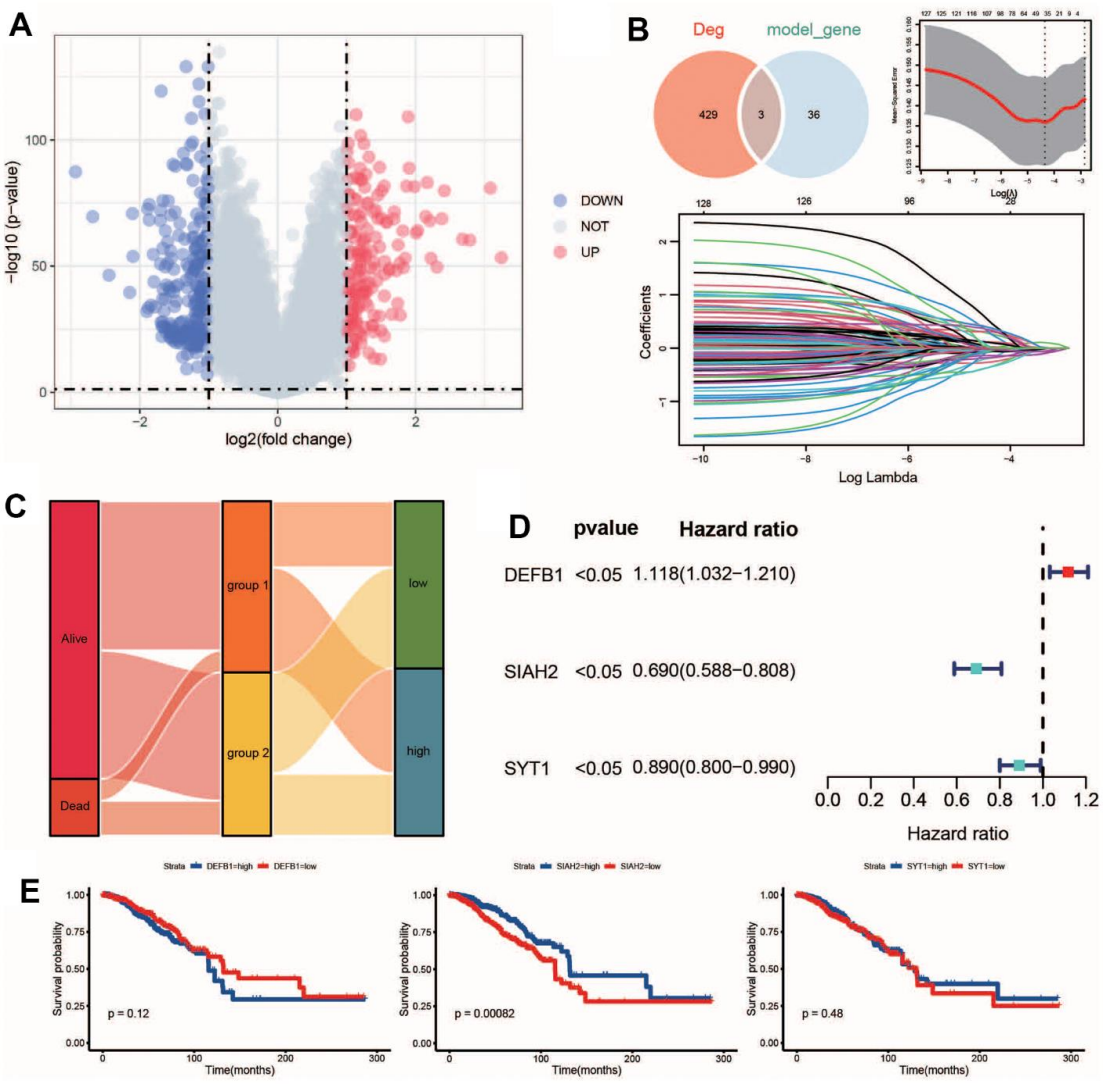
**Identification of key ADCP targets.** (**A**) Volcano plots and (**B**) LASSO regression model were used to identify differentially expressed genes (DEGs) and select key CRGs. (**C**) An alluvial diagram illustrated the changes in risk groups, ADCP groups, and survival state. (**D**) Univariate Cox regression analysis and (**E**) Kaplan-Meier curves evaluated the associations between infiltrate levels of ADCP targets and overall survival.

To explore the disparity in pathway enrichment analysis between the two clusters of ADCP in cohort A, GSEA was performed, with different immune infiltration patterns identified within the two subtypes. The enrichment histogram showed that cluster 1 significantly enriched hormone metabolism (including early estrogen response, late estrogen response) and inflammatory signal regulation pathways (allograft rejection, inflammatory response, kras signal and il6 jak stat3 signal, tnfa signal conduction through NFKB) (Figure 2A). At the same time, GSEA confirmed that there were differences in the immune pathways of ADCP clusters. The results showed that DEGs with higher expression levels in cluster 1 were significantly enriched in early estrogen response, allograft rejection pathway, inflammatory response, interferon response, kras signaling pathway and tnfa signal transduction through NFKB (Figure 2B). Given the strong correlation between ADCP subtypes and immune activity, the TME of the two clusters in cohort A was investigated (Figure 2C). Cluster 1 subtype is featured with high infiltration of Natural Kill cells (resting) and Macrophages, while cluster 2 subtype exhibits elevated infiltration levels of B cells, Plasma cells, T cells, Natural Kill cells, Dendritic cells, Mast cells and Neutrophils. Based on TCGA expression profile, the stromal score, immune score as well as ESTIMATE score of malignant tumor tissues were calculated through ESTIMATE algorithm. ESTIMATE produces a matrix score that measures the presence of tumor-associated matrices, as well as an immune score that reflects the level of immune cell infiltration. These scores are combined to produce an index called an ‘estimated score’ which provides a comprehensively estimation tumor purity. Compared with cluster 1, samples in cluster 2 also showed significantly higher estimated scores (Wilcoxon test, P < 0.05, Figure 2D). This trend was also observed for matrix scores as well as immune scores (Wilcoxon test, P < 0.05). Additionally, we studied the association between the two subtypes and major histocompatibility complex (MHC) and T cell stimulators. In addition to HLA-C, the expression levels of MHC as well as T cell stimulators exhibited a tendency to be higher in cluster 2 (Figure 3E, 3F).

### Recognition of key ADCP genes

Using the limma algorithm, a total of 432 degs (Differentially expressed gene) between group1 and group2 are identified under the filtering threshold of FDR q-value < 0.01 and the absolute value of logFC > 1 (Figure 3A and Supplementary Table 2). Prognostic models were constructed for 1006 BC patients with OS information in cohort A. LASSO Cox regression analysis was utilized to determine the best prognostic features based on 130 survival-related ADCP genes. After the variables were included in the LASSO Cox regression model with the smallest lambda, the genes of 39 ADCP-related features were chosen to construct the ADCP-related risk scoring model. Three key ADCP genes (DEFB1, SIAH2 and SYT1) by overlapped DEGs from cohort A and the model ADCP genes were identified (Figure 3B). The relationship of the expression levels with 3 ADCP-related signatures together with OS are also presented in the forest plot (Figure 3D). The expression of three key ADCP genes is of significance for survival (Figure 3E).

### Multidimensional analysis of key ADCP genes

**Figure 4:**
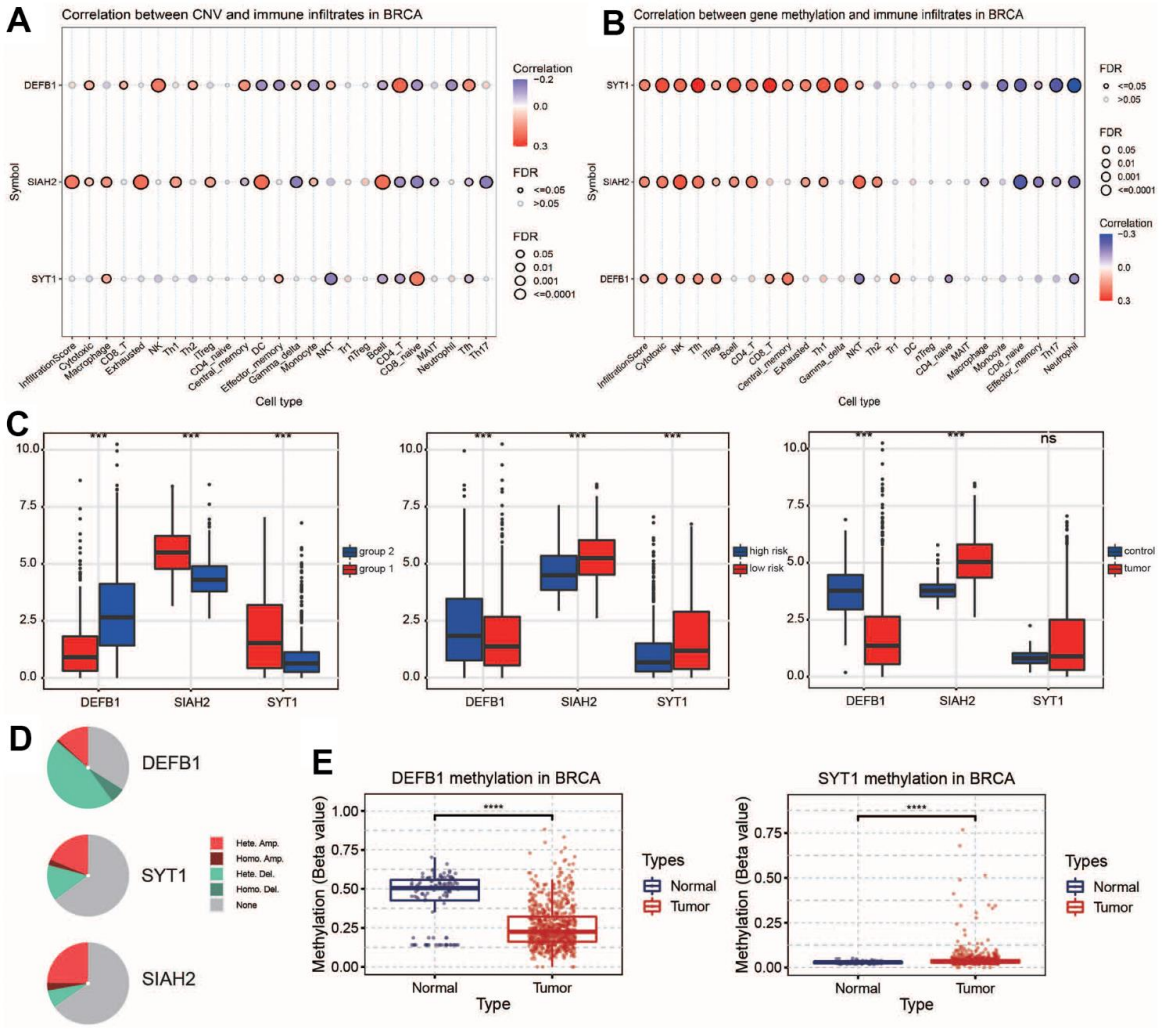
**Validation of key ADCP genes in expression level.** (**A**, **B**) Correlation analyses were conducted between CNV/methylation and immune infiltrates in BC. (**C**) Expression levels and (**D**, **E**) CNA percentages of key ADCP genes were compared between ADCP groups, risk groups, and cancer types. Methylation levels of key ADCP genes in BC and normal samples were also examined. Statistical significance at the level of ns ≥ 0.05, *** <0.001 and **** <0.0001.

We studied the relationship of CNV with immune infiltration in BRCA. Additionally, we explored the association of gene methylation with immune infiltration. CNV and methylation of key ADCP genes were closely associated with the infiltration of key immune cells including T and B cells (Figure 4A, 4B). At the same time, based on cohort A, the gene expression levels of 3 key ADCP genes were analyzed. In the data set, only DEFB1 was down-regulated in group1, while SIAH2 and SYT1 were up-regulated in group2 (Wilcoxon test, P > 0.05). Only DEFB1 was up-regulated in the high-risk group, while in the low-risk group, SIAH2 together with SYT1 were down-regulated (Wilcoxon test, P > 0.05). Only DEFB1 was up-regulated in the tumor group; in contrast, SIAH2 and SYT1 were down-regulated in the low-risk group (Wilcoxon test, P > 0.05, Figure 4C). Among the three key ADCP genes, the frequency of copy number alterations (CNA) of SIAH2 was higher compared to that of the others. Specifically, CNA deletions were predominant among all types (Figure 4D). Methylation analysis showed that the beta value of SIAH2 was higher in the tumor group than in the normal group, and DEFB1 was the opposite (Figure 4E). It is well known that gene expression is negatively correlated with the level of methylation, while CNA has a positive effect on gene expression. In summary, CNA and methylation may be may be important in the up-regulation of SYT1 expression in BC.

### ADCP-related signatures for the prognostic prediction of BC

**Figure 5:**
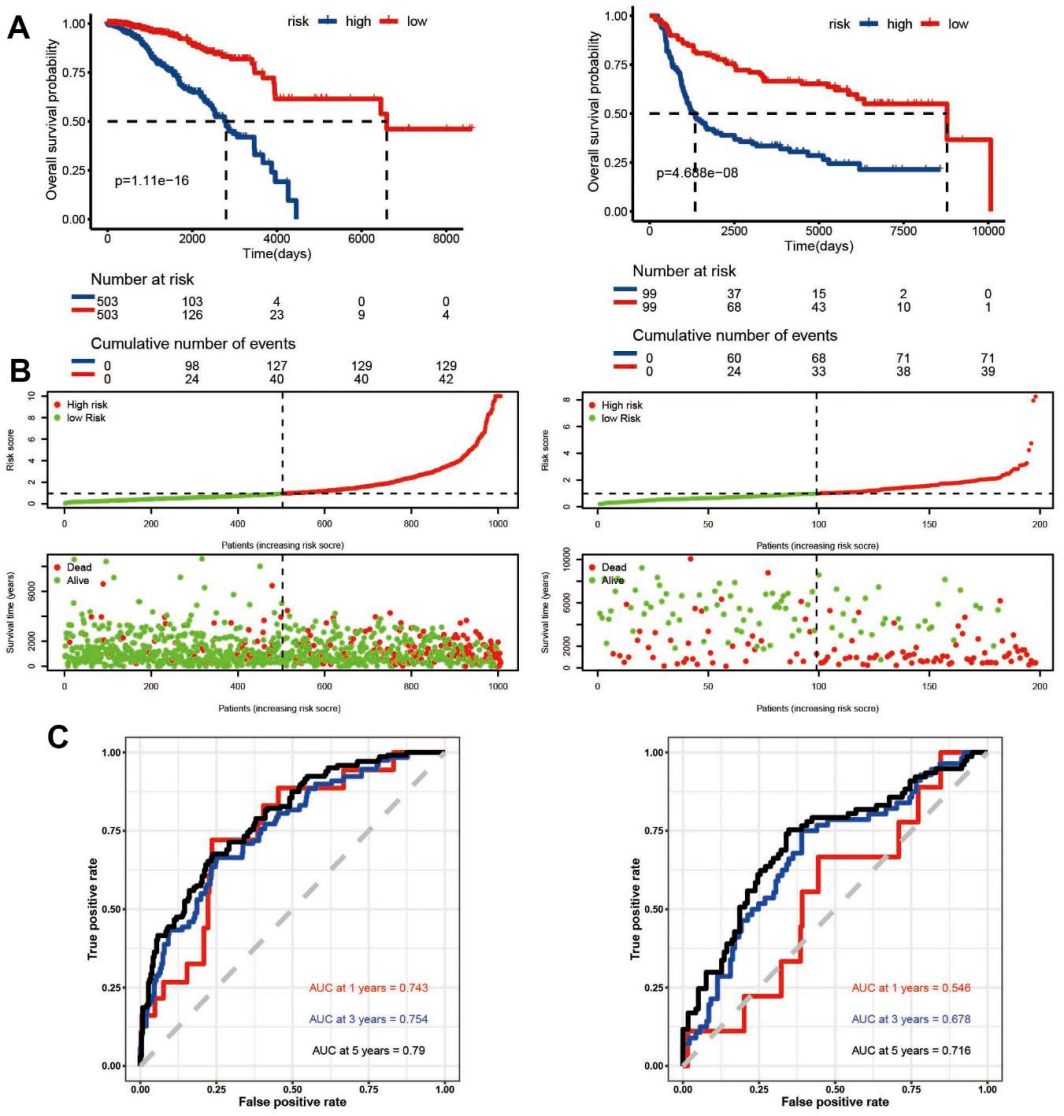
**Survival evaluation of ADCP-related risk scoring model in TCGA and METABRIC datasets.** (**A**) Kaplan-Meier survival curves, (**B**) patient subgrouping based on risk scores, and (**C**) ROC curves assessed the performance of the ADCP-related risk scoring model in predicting overall survival at 1, 3, and 5 years.

LASSO algorithm was used to establish a risk model. Finally, 39 genes related to prognosis were identified, and used to construct models based on risk scores using the training (n = 1006) and test (n = 1980) data sets of cohorts A and B patients, respectively. Survival analysis showed that a higher risk score of the training and the test sets was related with a lower survival rate (p < 0.0001) (Figure 5A, 5B). A time-varying ROC curve was generated for the assessment of the sensitivity of the model. The 3-year, 5-year, and 10-year AUCs were found to be 0.743,0.754, and 0.79 in the training set, respectively (Figure 5C); in contrast, they were 0.546,0.678, and 0.716 in the test set, respectively (Figure 5C).

### The ADCP group served as an independent prognostic factor in BC

**Figure 6:**
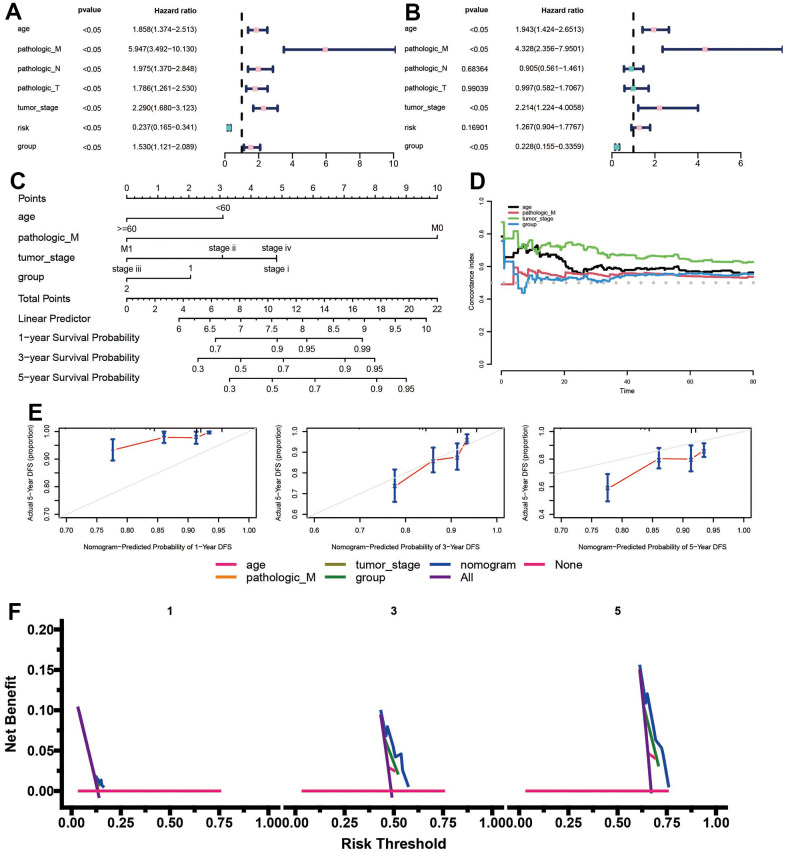
**A nomogram was developed to predict the probability of 1-, 3-, and 5-year overall survival (OS) in the training cohort.** (**A**, **B**) Univariate and multivariate analyses were conducted, including the ADCP-related risk scoring model and clinical factors. (**C**) The comprehensive nomogram provided predictions for BC patients’ OS probabilities in the TCGA dataset. (**D**) The time-dependent c-index plot compared the performance of the nomogram with other clinical factors. (**E**) Calibration plots assessed the accuracy of the nomogram’s predictions for 1-, 3-, and 5-year OS in the TCGA cohort. (**F**) Decision curve analysis evaluated the clinical utility of the nomogram and other factors for 1-, 3-, and 5-year risk assessment.

Since ADCP genes are significantly associated with high malignancy and advanced tumors of BC, univariate and multivariate Cox regression analysis were carried out to determine the prognostic significance of ADCP genes for BC patients. ADCP group, age, TNM stage, stage together with risk score were included as covariates. Results showed that ADCP group, age, TNM stage, stage as well as risk score were independent prognostic factors for BC patients (Figure 6A, 6B). we constructed a nomogram by combining independent prognostic factors, serving as a clinically relevant quantitative method tool for predicting the mortality of individual BC patient (Figure 6C). Based on the c-exponential curve of different variables over time in the TCGA cohort, nomogram performed best compared to other single factors (Figure 6D). Add up the scores of each prognostic parameter and assign a total score to each patient. The higher the total score, the worse the prognosis of patients. The modal diagram has similar performance to the ideal model (Figure 6E). In addition, the DCA curve also indicated that the nomogram had good stability and reliability net benefit curve in age compared with other clinical factors (Figure 6F).

### ADCP groups for the prediction of the chemotherapeutic response

**Figure 7:**
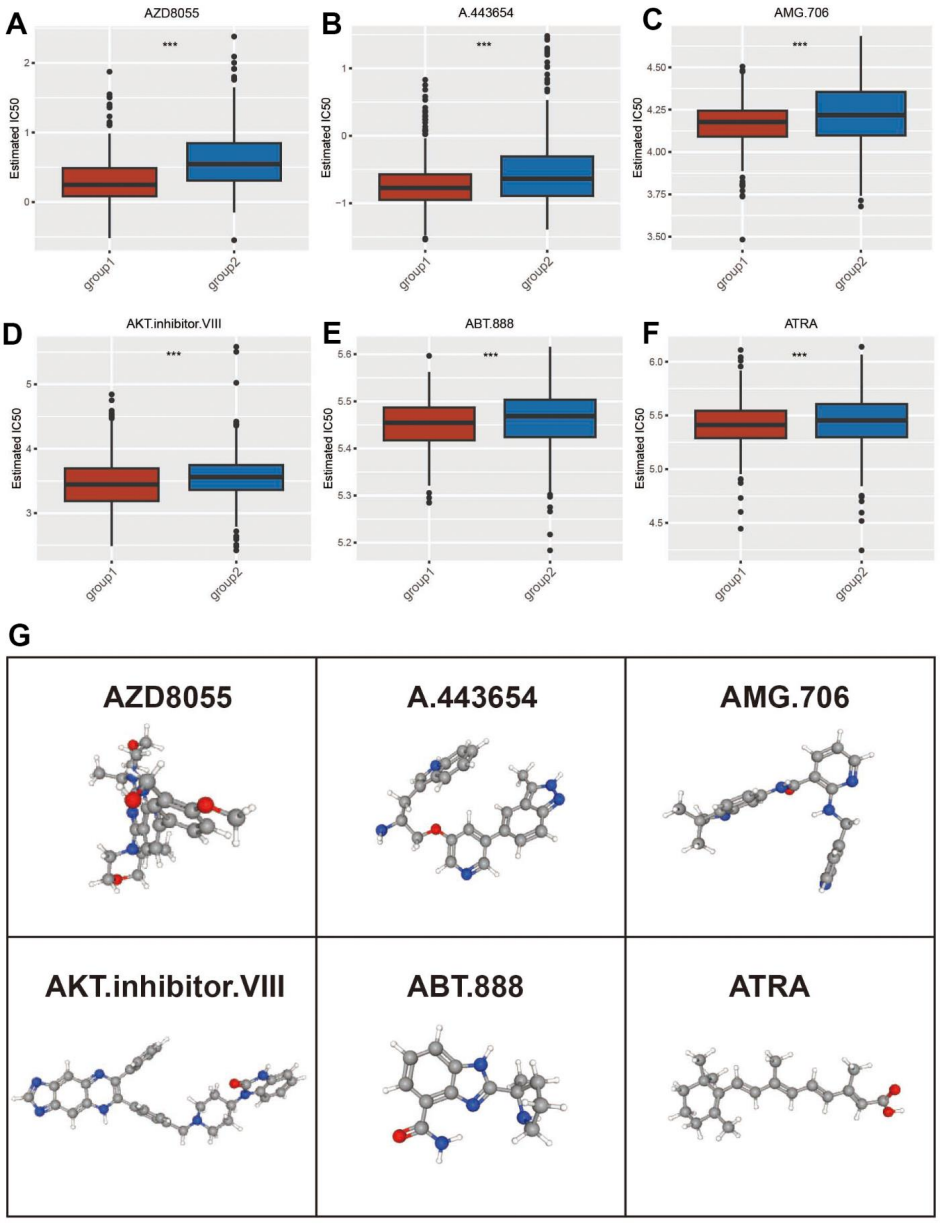
**The ADCP-related groups were evaluated for their ability to predict therapeutic benefit.** (**A**–**F**) The chemotherapy response of two ADCP-related groups to six common chemotherapy drugs was analyzed. (**G**) The 3D structure tomographs of six candidate small-molecule drugs for BC were examined. Statistical significance at the level of *** <0.001.

We evaluated the chemotherapy response and drug resistance of patients in the ADCP group. Figure 7 shows the sensitivity of two ADCP subtypes to six anticancer drugs (AZD8055, A.443654, AMG.706, AKT.inhibitor.VIII, ABT.888, ATRA). Results showed that the IC50 level of group2 was higher compared to group1 (Figure 7A–7F), and small molecule drugs with therapeutic effects on BC could be found according to the results of drug sensitivity. Three-dimensional structural tomography of AZD8055, A.443654, AMG.706, AKT.inhibitor.VIII, ABT.888 and ATRA was found in PubChem (Figure 7G).

### Gene expression level verification via qRT-PCR and WB

**Figure 8:**
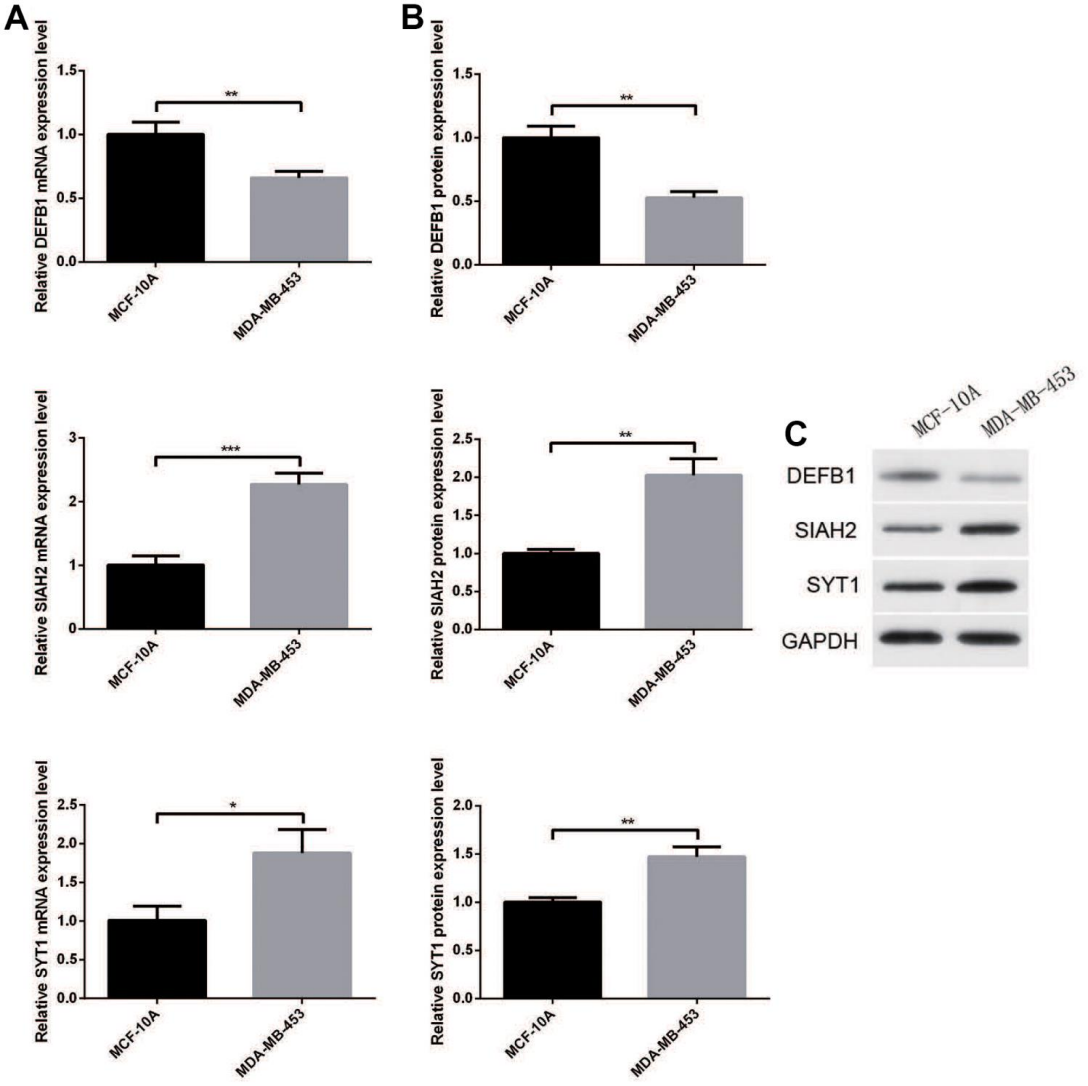
(**A**) RT-qPCR and (**B**, **C**) Western blotting analysis of DEFB1, SIAH2, and SYT1 expression in BC cell lines and adjacent cell lines. Statistical significance at different levels was reported for the results. Statistical significance at the level of * <0.05, ** <0.01, and *** <0.001.

We verified the mRNA and protein levels of DEFB1, SIAH2 and SYT1 in BC cell lines and adjacent cell lines by qRT-PCR and WB. Results of qRT-PCR is shown in Figure 8A. In contrast with the normal control, DEFB1 level in BC cell line MDA-MB-453 was lower, but the SIAH2 and SYT1 levels were higher. The protein expression levels of the three key ADCP-genes based on WB analysis were consistent with the results of qRT-PCR experiments (Figure 8B, 8C).

## DISCUSSION

Traditional cancer treatment methods such as chemotherapy are non-selective and increase the energy of the immune system, leading to serious side effects and tumor recurrence [30]. Since most tumor-associated antigens are abundantly expressed in cancer cells and some normal cells, it is a challenge for the human immune system to produce cancer antigen-targeted antibodies in cancer patients [31]. The rise of ADCP-related research has revolutionized cancer treatment. Changes, through ADCP-related targets can obviously improve the OS rate of cancer patients [32–36].

Despite the extensive understanding of the intercellular process of phagocytosis, specific genetic changes within cancer cells that drive / expel ADCP remains have not been fully determined. Recently, Kamber et al. [37] identified a collection of previously unknown cancer-intrinsic genes that play a role in ADCP based on an unbiased CRISPR/Cas9 screen. In this investigation, Unsupervised consistent clustering was adopted to cluster gene expression patterns of Immune checkpoint, immune microenvironment, inflammation related molecules, immune cells as well as stromal elements derived from BC datasets and ADCP cancer-intrinsic genes. In this study, we successfully discovered a new ADCP-related BC category comprising 51.2% of BC patients. The ADCP-BC group, in contrast to the other group, showed lower immunological and stromal enrichment scores, suggesting a reduced presence of immune cells and stromal components. The ADCP-BC group exhibited distinct characteristics, including obvious immune cell infiltration, increased expression of multiple inflammatory genes and HLA family gene. At the same time, the three ADCP-related targets identified also showed significant differences in different groups and cancer-adjacent samples. This shows a potential advantage for them as targets for clinical treatment. Our findings have significant translational implications in the field. In addition to its prognostic value, our identified signature demonstrates a notable association with drug sensitivity, emphasizing its potential clinical relevance and applicability in therapeutic interventions. Based on these findings, it could be inferred that the ADCP group may benefit more from AZD8055, A.443654, AMG.706, AKT.inhibitor. VIII, ABT.888 and ATRA than from ICI, suggesting the combination of ZD8055, A.443654, AMG.706, AKT. inhibitor. VIII, ABT.888 and ATRA with ICI may be as potential therapeutic drug.

DEFB1 has not been specifically reported in the context of phagocytosis. However, it has received significant attention within the field of cancer research. Defensins form a family of microbicidal and cytotoxic peptides made by neutrophils. Members of the defensin family are highly similar in protein sequence. This gene encodes defensin, beta 1, an antimicrobial peptide implicated in the resistance of epithelial surfaces to microbial colonization. This gene maps in close proximity to defensin family member, defensin, alpha 1 and has been implicated in the pathogenesis of cystic fibrosis. Results of a previous study suggested that the DEFB1 gene, which encodes human ß-defensin-1 (HBD-1), plays a role in innate immune responses and may act as a potential tumor suppressor in urological cancers [38]. Furthermore, it has been observed that DNA methylation pattern within non-CpG island promoter region of DEFB1 can influence epigenetic silencing of DEFB1 in tumor cells [39]. In colorectal cancer, SIAH2 has been identified as an oncogene [40]. SIAH2 (Siah E3 Ubiquitin Protein Ligase 2) is a Protein Coding gene. Diseases associated with SIAH2 include Dyskeratosis Congenita, Autosomal Dominant 3. Among its related pathways are Class I MHC mediated antigen processing and presentation and Nervous system development. Gene Ontology (GO) annotations related to this gene include ligase activity and transcription corepressor activity. It promoted various aggressive behaviors of colorectal cancer cells, such as proliferation, migration, invasion, as well as colony formation [41]. Interestingly, high mRNA levels of SIAH2 may be correlated with elevated Estrogen Receptor (ER) mRNA levels and improved progression-free survival (PFS) after initial tamoxifen [42, 43]. Studies have shown that cytoplasmic proteins interact with SYT1 on the endoplasmic reticulum and then are spatially localized in the SEC22B + vesicles of liver cancer cells. This gene encodes a member of the synaptotagmin protein family. The synaptotagmins are integral membrane proteins of synaptic vesicles that serve as calcium sensors in the process of vesicular trafficking and exocytosis. The encoded protein participates in triggering neurotransmitter release at the synapse in response to calcium binding. Therefore, SEC22B on the vesicles is secreted on the PM by Q-SNAREs (SNAP23, SNX3 and SNX4) [44]. In addition, inhibition of the interaction between protein kinase Cδ (PKCδ) and SYT1 by PKCδ antibody can reduce the secretion and tumorigenicity of PKCδ. PKCδ is a cytoplasmic protein specifically secreted by hepatocellular carcinoma [44]. The results revealed the significance of ER-PM contact sites in facilitating the secretion of cytoplasmic protein, establishing a foundation for targeted therapies of liver cancer [45]. As mentioned above, chemotherapy drugs as well as targeted drugs are the primary therapeutic approaches for treating BC [46]. In our study, a significant increase of ADCP targets was observed in BC patients, suggesting that ADCP targets can serve as an indicator for predicting the efficacy of BC patients. Furthermore, the IC50 values of certain chemotherapeutic drugs was compared with those of targeted drugs between ADCP groups, and results showed that BC patients in the low ADCP group may present with a more favorable response to the drugs.

This study had certain limitations. The cohorts were obtained from high-throughput sequencing platforms in distinct public datasets, making the presence of intratumor/intrapatient tumor heterogeneity inevitable. Previous investigations have found that tumor heterogeneity might exert an influence on the efficacy of immunotherapy or chemotherapy. Unfortunately, owing to limitations in the available data, we were obliged to overlook the remarkable heterogeneity observed in cases of BC. Secondly, although immune interaction and survival effects associated with the inflammation pathway and ADCP-targets were observed among BC patients, their underlying biological/medical mechanisms remain obscure. Thus, it is imperative to conduct extensive large-scale prospective studies along with functional/mechanistic experiments to validate and elucidate the effect of the inflammation pathway on BC. Thirdly, the median cutoff of survival-related ADCP genes was adopted to stratify BC samples into the high and low survival-related groups. Despite that, the optimal cutoff for the survival-related ADCP genes may be a superior stratification strategy for BC patients. Lastly, due to incomplete clinicopathological information, we organized and adjusted certain clinical data for survival and Cox regression analyses, which may introduce the possibility of potential biases and uncertainties in determining the prognostic significance of the ADCP-related group.

The demand for precision medicine in cancer treatment is urgent. Our article has hints for the classification and targets of breast cancer. These targets can help the development of chemotherapy drugs in computer-aided drug design. Our research content expands the research of ADCP in the field of breast cancer, and also provides reference for other types of cancer. The research on ADCP for other cancers can learn from this research method.

## CONCLUSIONS

In summary, we identified a subtype and key pathogenic factor associated with antibody-dependent cellular phagocytosis in the pathogenesis of breast cancer, accounting for about 51.2 % of BC patients, showing better therapeutic effect on tumor drug treatment and unique immune molecular characteristics of tumor immune microenvironment. Our results offered novel insights into the molecular mechanisms underlying ADCP therapy, contributing to the development of personalized immunotherapy strategies.

## Supplementary Material

Supplementary Table 1

Supplementary Table 2
